# Nurses’ perceptions, experiences, and practices regarding human papillomavirus vaccination: results from a cross-sectional survey in Montana

**DOI:** 10.1186/s12912-023-01379-6

**Published:** 2023-06-19

**Authors:** Juthika Thaker, Alexandria N. Albers, Sophia R. Newcomer

**Affiliations:** 1 School of Public and Community Health Sciences, The University of Montana, 32 Campus Drive, Skaggs 173, Missoula, MT 59803 USA; 2 Center for Population Health Research, The University of Montana, 32 Campus Drive, Missoula, MT 59812 USA

**Keywords:** Nursing practice, Rural populations, HPV vaccination, Evidence-based strategies, Cancer prevention

## Abstract

**Background:**

Nationally, much of the focus on improving human papillomavirus (HPV) vaccine uptake has been on effective strategies that physicians use to promote vaccination. However, in large, predominately rural states like Montana, nurses and medical assistants play critical roles in immunization services delivery, and their viewpoints are imperative in designing strategies to increase vaccination rates. We conducted a cross-sectional, descriptive study to determine nurses’ perceptions, experiences, and practices regarding human papillomavirus vaccination in a rural and medically underserved region of the United States.

**Methods:**

We designed, pilot-tested, and disseminated an online survey instrument to nurses and medical assistants working in clinics participating in the Vaccines for Children program in Montana. The online surveys were administered from November 2020 to March 2021. Survey questions focused on clinic vaccination practices, respondents’ perceptions of the HPV vaccine, perceived barriers to vaccine uptake, and general opinions on potential strategies to improve HPV vaccination rates.

**Results:**

We analyzed data from 227 respondents. Overall, 90% of nurses strongly agreed or agreed that the HPV vaccine is important and had confidence in the vaccine’s safety. More nurses reported experiencing greater parental vaccine refusal or delay for male patients regardless of age. About 53.7% of nurses reported that their clinics had reminder/recall systems to encourage parents to bring their children for vaccination. Nurses identified misinformation from social media, infrequent wellness visits, and vaccine safety concerns as barriers to HPV vaccine uptake.

**Conclusions:**

Study findings identified several promising initiatives to accelerate vaccination in primarily rural states like Montana, including promoting widespread adoption of reminder/recall systems, training nurses in evidence-based techniques to provide strong vaccine recommendations, and leveraging social media to disseminate consistent messages about the HPV vaccine recommendations for both sexes and its role in cancer prevention.

**Supplementary Information:**

The online version contains supplementary material available at 10.1186/s12912-023-01379-6.

## Introduction

Human papillomavirus (HPV) is the most common sexually transmitted infection in the United States, with 13 million new cases emerging every year [1]. Although most HPV infections are asymptomatic and self-limiting, persistent HPV infection can cause cervical cancer in women as well as other anogenital cancers, oropharyngeal cancer, and genital warts in men and women [2, 3]. The U.S. Advisory Committee on Immunization Practices (ACIP) routinely recommends HPV vaccination at 11–12 years of age; however, vaccination can be given as early as 9 years of age [4]. If the first vaccine dose is received before the 15th birthday, then two vaccine doses are required to complete the series; otherwise, three doses are needed for series completion [4].

Rural communities in the U.S. face a disproportionate burden of health disparities due to factors including barriers to accessing primary care services; higher rates of un- or under-insurance; lower health literacy, and vaccination rates; and a shortage of pediatricians [5–8]. Indeed, pediatricians contribute to higher vaccine uptake in their communities. For example, pediatricians more often administered HPV series to their patients and reported higher confidence in their ability to address HPV vaccine concerns when compared to family medicine practitioners [9–11]. Rural areas have also been associated with negative parental attitudes about the HPV vaccine, greater safety concerns about the HPV vaccine and higher incidence and mortality from HPV-caused cancers [12–14]. In Montana, a large and primarily rural state, the HPV vaccine series completion rate in 2020 was 54.4% for adolescents ages 13–17 years, as reported by the Centers for Disease Control and Prevention’s National Immunization Survey-Teen, below the Healthy People 2030 goal of 80% [15, 16]. Furthermore, for the years 2015–2019, the proportion of rural adolescents in Montana who received at least one dose of the HPV vaccine (56.0%) was 11.9% points lower when compared to Montana adolescents living in more urban areas (67.9%), indicating a pronounced urban-rural disparity in vaccine uptake [16].

In a study by Newcomer et al., physicians and public health stakeholders in Montana identified greater parental/patient informational needs and limited time for vaccine discussions as barriers to HPV vaccination [17]. Non-physician healthcare providers, like nurses, could help bridge this gap and serve as champions and promoters of the HPV vaccine. Adolescents residing in rural areas are more likely to use non-traditional facilities like public health clinics for their immunization needs [5]. In public health facilities, nurses and medical assistants regularly interact with children and adolescent patients and their parents/guardians. Past studies have consistently shown that absent or weak recommendations from healthcare providers drive poor vaccine uptake [18–20]. Thus, designing interventions to ensure that healthcare professionals other than pediatricians are familiar and confident with adolescent vaccine recommendations is crucial in predominately rural states like Montana. Nurses play a significant role in vaccine delivery services by educating parents and patients on vaccines, alleviating parental concerns and vaccine hesitancy, and are well-positioned to facilitate coordination efforts within their practices [3, 21, 22]. Although nurses serve as primary stakeholders in developing and implementing health promotion initiatives, there are limited published data focused on understanding nurses’ perspectives on HPV vaccination [22–24].

To address this research gap, we designed a cross-sectional, descriptive study to determine Montana nurses’ and medical assistants’ perceptions, experiences, and practices in providing adolescent immunization services, with a focus on HPV vaccination. Our study findings identified recommendations that can inform initiatives to effectively engage nurses in improving HPV vaccination rates in states with high rural and medically underserved populations.

## Methods

### Sample

The sample population for this study consisted of registered nurses (RNs), advanced practice registered nurses (APRNs), and medical assistants in Montana currently employed at a facility that participated in the federal Vaccine for Children (VFC) program. The VFC program provides childhood and adolescent vaccines to enrolled providers for immunizing eligible children through 18 years of age at no cost [25]. Since its implementation in 1994, the Centers for Disease Control and Prevention (CDC) has estimated that the VFC program has been instrumental in saving about 295 billion dollars in direct costs by 2013 [26]. Over 90% of facilities that provide immunization services to children in Montana participate in VFC.

### Design

An online survey instrument was developed and administered using the electronic database REDCap, hosted by the University of Washington Institute of Translational Health Sciences [27]. The survey questionnaire was developed based on a review of existing literature on vaccine attitudes and previous CDC-funded surveys of primary care physicians’ perspectives on HPV vaccinations [9–11]. The final survey tool comprised five sections. The first section collected information on the participants and their medical roles and responsibilities. The second section was designed to learn more about clinic vaccination practices, including the use of reminder/recall systems. Reminders alert patients about vaccinations that will be due in the future and recall messages are used to inform patients about the vaccinations that are overdue [28, 29]. In the third section, we included questions on nurses’ perceptions regarding the HPV vaccine, their experiences with parental awareness and refusal or deferral of the HPV vaccine, and perceived barriers to adolescents receiving the HPV vaccine. Section four had questions on the nurses’ vaccine attitudes, beliefs, and perceptions of the effectiveness of strategies for improving the HPV vaccination rates; and the last section contained a few demographic questions. (Table 1) Participants could utilize comment boxes throughout the survey to provide additional open-ended feedback.

The survey instrument was pre-tested and modified based on cognitive interviews with a convenience sample of six nurses and one medical assistant. Survey pre-testing was conducted on a virtual platform. Cognitive interviewing techniques (Think-aloud approach and Verbal probing) were employed to walk nurses through the survey and collect their feedback on the comprehensibility of the questions and overall survey design [30]. Most comments were positive, with cognitive interview participants emphasizing the need for a state-wide survey on this topic. Based on pre-testing, we estimated that most participants would be able to complete the survey within 12–15 min. Nurses and medical assistants who participated in the pre-testing were given a $20 gift certificate for their time and input and were not excluded from participating in the survey. Based on the feedback received from cognitive interview nurse participants, we modified response options, added response options, and rephrased certain questions to improve comprehensibility; eliminated one question that nurses deemed appropriate for “administrative” staff; and adapted demographic questions to resemble the ones asked in the national immunization surveys to improve recall. The final survey instrument had a total of 23 content questions and 6 demographic questions.

**Table 1:** Example survey questions for each section included in the survey instrument

Survey Sections	Example Questions
Participant and practice characteristics (9 items)	Approximately what percentage of patients that you see are eligible for the Vaccines for Children (VFC) program? • *Less than 25%* • *25-49%* • *50-75%* • *More than 75%* • *Not sure*
Clinic vaccination practices (7 items)	How does your clinic contact patients later to return for their additional HPV vaccine doses? • *Phone Call* • *Text Message* • *E-mail* • *Paper letter/ Postcard*
Nurses’ experiences and perceptions of parental vaccine acceptance or hesitancy (5 items)	In your experience, what percentage of parents/guardians refuse or defer the HPV vaccine in each of the following age groups and gender? • *Less than 10%* • *10-25%* • *26-50%* • *More than 50%* • *Don’t Know/ Not Sure*
Nurses’ attitudes and beliefs regarding HPV vaccination (2 items)	In your opinion, how effective do you think the following strategies would be for increasing rates of human papillomavirus (HPV) vaccination among older children and adolescents? *Emphasizing cancer prevention when discussing the HPV vaccine with parents and older children and adolescents* • *Very Effective* • *Somewhat Effective* • *Neutral* • *Not Effective* • *Don’t Know/Not Sure*
Other demographic questions (6 items)	Which of the following age groups do you belong to? • *Less than 20 years* • *21–30 Years* • *31–40 years* • *41–50 years* • *51–60 years* • *≥ 60 years*

### Data collection procedures

A list containing the email addresses of VFC coordinators was obtained by the study team from the Montana Department of Health and Human Services Immunization Program section. We sent an email containing a short study description, the study team’s contact information, and the survey link to VFC contacts in 250 different clinic settings across Montana in November 2020. The VFC coordinators were requested to distribute the survey among all nurses and medical assistants working in immunization services in their facilities. VFC coordinators who were practicing nurses and provided immunization services to adolescents were encouraged to take the survey as well. Two additional email reminders were sent to VFC coordinators after the initial survey invitation at equal intervals of 30 days, after which the survey was closed in early March 2021. After the study closure, three participating nurses were randomly selected to receive a $30 gift card.

### Statistical analysis

All statistical analyses were performed using SAS version 9.4 (SAS Institute, Inc, Cary, NC). We have presented descriptive statistics, i.e., frequencies and percentages for nurses’ responses to each question that was included in the questionnaire. We retained data from partial as well as completed survey questionnaires for final analysis.

### Ethical considerations

Participants were required to affirmatively indicate their willingness to participate in the study by clicking a box (or marking an X) before proceeding into the survey as per The University of Montana’s Statement of Confidentiality for online surveys. Participants were informed that their responses would be kept confidential and study findings will be reported on an aggregate basis. The study was approved by the University of Montana Institutional Review Board. The IRB reviewed the study protocol and supporting documents and approved the study under the exempt category of review (IRB Protocol No.: 146 − 20).

## Results

We received a total of 309 responses, and 296 nurses provided their consent to participate in the survey. Of these 296 nurses, n = 6 reported that they did not currently work as either a nurse or medical assistants in the state of Montana and n = 20 reported that they were not involved with adolescent immunization services; these respondents were excluded. Out of the remaining 270 respondents, n = 16 respondents were further excluded because they did not provide their nursing or medical credential and n = 27 respondents were excluded as they reported not currently working in direct patient care. The final analytic sample consisted of 227 respondents.

(Fig. 1).

**Fig. 1:**
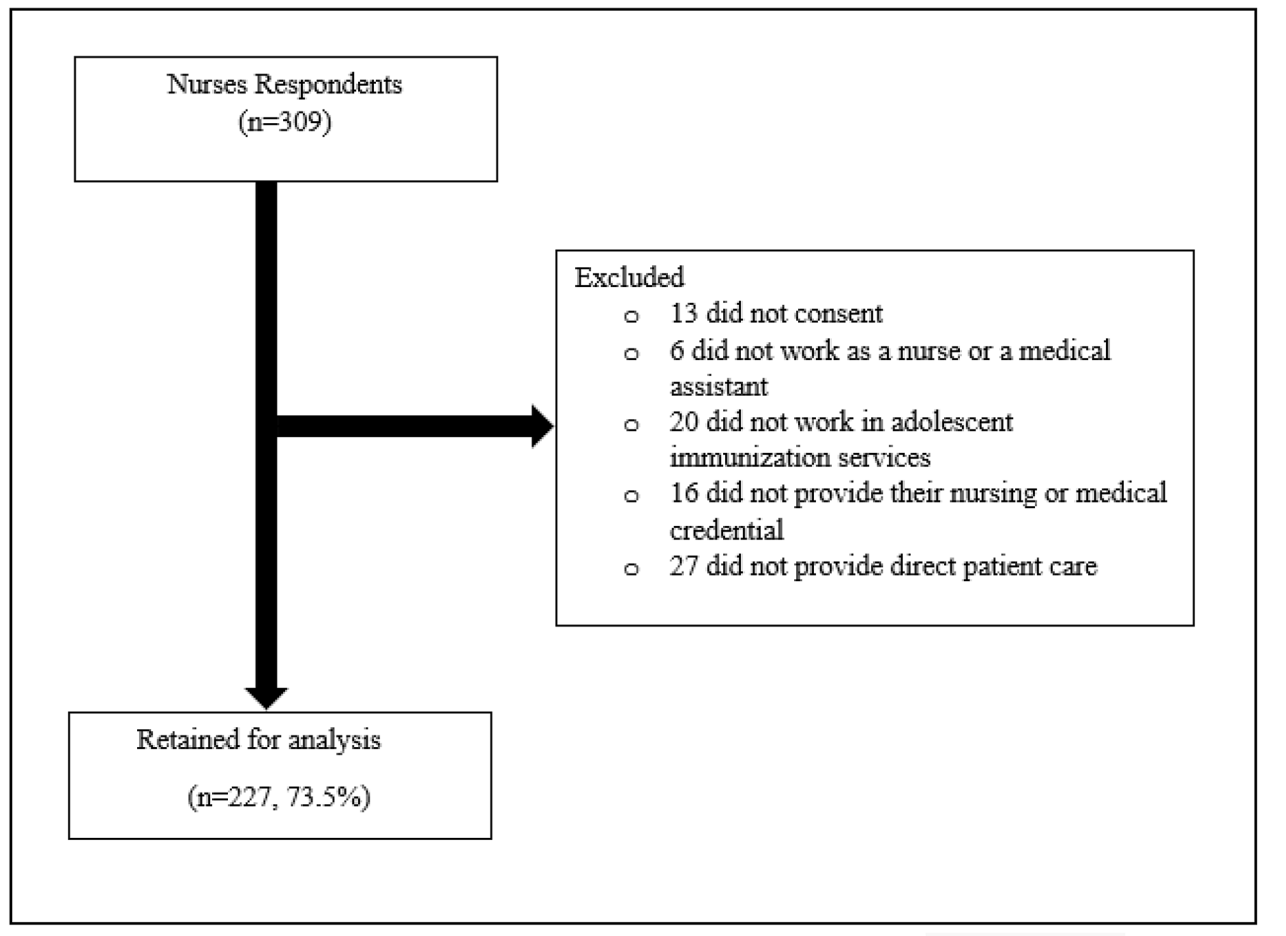
Flow diagram detailing survey eligibility

### Characteristics of respondents

Of the 227 eligible respondents, most (n = 127, 55.9%) were registered nurses or advanced practice registered nurses, 26.0% (n = 59) were medical assistants, and 17.6% (n = 40) were licensed practical nurses. A majority (n = 180, 94.2%) of the respondents were female and identified themselves as being white (n = 175, 77.1%). About 4.0% (n = 9) of the respondents identified themselves as American Indian or Alaska Native. About 27.4% (n = 52) of the respondents belonged to the age group of 41–50 years, followed by 23.2% (n = 44) of the respondents who reported being in the age group 51–60 years. Approximately 33.0% (n = 63) of the participants reported working as a nurse or a medical assistant for more than 20 years, 18.2% (n = 35) for about two to six years, and 17.0% (n = 33) for around six to ten years. Only 7.0% (n = 14) of the nurses in our analytic sample had less than two years of experience working as nursing professionals. (Table 2)

**Table 2:** Survey Respondent and Practice Characteristics

	n *(N = 227)*	%^β^
**Nursing credentials**		
Registered Nurse (RN/APRN) Licensed Practical Nurse (LPN) Medical Assistant or Other	127 40 60	55.9% 17.6% 26.4%
**Age, years**		
21–30 years 31–40 years 41–50 years 51–60 years ≥ 61 years Prefer not to answer/ Missing	36 41 52 44 16 38	15.9% 18.1% 22.9% 19.4% 7.1% 16.7%
**Sex**		
Male Female Prefer not to answer/ Missing	9 180 38	4.0% 79.3% 16.7%
**Clinic setting**		
Public health department-operated clinic Private practice or a hospital/university-based clinic Other* Missing	44 82 90 11	19.4% 36.1% 39.7% 4.9%
**Practice location**		
Non-Metropolitan Statistical Area Micropolitan Statistical Area Metropolitan Statistical Area Missing	85 58 47 37	37.4% 25.6% 20.7% 16.3%
**Estimated number of 9-17-year-old patients seen in a typical week**		
≤ 5 patients 6–20 patients > 20 patients Not Sure	118 74 23 12	52.0% 32.6% 10.1% 5.3%
Estimated percentage of 9-17-year-old patients eligible to receive vaccines under the VFC** program		
< 25% 25-49% 50-75% > 75% Not Sure Missing	24 58 68 35 27 15	10.6% 25.6% 30.0% 15.4% 12.0% 6.6%

Column percentages do not always total to 100% due to rounding of the values, * includes a community health center, rural health clinic, migrant health center, Indian Health Service (IHS)-operated center, Tribal health facility, or urban Indian health care facility, Military health care facility (Army, Navy, Air Force, Marines, Coast Guard), WIC clinic, school-based clinic, and any other clinic type, ** VFC indicates Vaccine for Children federal program

β Percentages may not add to exact 100 due to rounding

### Practice characteristics

While 36.1% (n = 83) of respondents either worked in an independent private clinic or a hospital-based clinic, 19.4% (n = 45) worked at a public health department, and the rest of the respondents (n = 90, 39.7%) either worked at a community health center, a rural health clinic, a school-based clinic, or a different type of immunization clinic. About 5.0% (n = 11) of respondents did not report their clinic setting. About half of the respondents (n = 118, 52.0%) examined five or fewer 9-17-year-old patients in a typical week. While all respondents were involved in providing immunization services to adolescents, over 85.0% (n = 200) of respondents reported recommending vaccines to adolescents and their parents or caregivers and interacting with them to answer vaccine-related questions. About two-thirds (n = 148, 65.2%) of the respondents reported scheduling clinic visits for immunizations, and about 58.2% (n = 132) reported overseeing vaccine ordering and managing vaccine inventory at their clinics. About 50.0% (n = 103) of the respondents reported that over half of the patients visiting their facility were eligible to receive free vaccines under the VFC program. (Table 2)

### Use of reminder/recall (R/R) systems for HPV vaccination delivery

About 52.0% (n = 109) of respondents reported using some form of reminder/recall (R/R) processes at their clinics to identify and contact parents/caregivers of adolescents who are due or overdue to receive recommended immunizations. Of those that use some form of R/R at their facilities, about 28.9% (n = 30) of respondents reported that staff availability dictated how often they were able to generate them, and about 25.0% (n = 26) of the nurses responded being able to generate the R/R lists monthly. The most common mode of R/R delivery was by phone (n = 86, 38%), a paper letter or a postcard (n = 70, 30.8%), or a text message (n = 23, 10.1%).

Specific to R/R processes for completing the multi-dose HPV vaccine series, most respondents reported that parents were told when they needed to return for the second dose at the initial vaccine appointment (n = 144, 63.4%) or that the subsequent immunization visit was scheduled during the initial appointment (n = 126, 55.5%). Only 26.9% (n = 61)of respondents reported that their clinics proactively reached out to parents or patients to remind them to return for additional HPV vaccine doses, and 5.3% (n = 12) of nurses reported that their clinics had no process to remind adolescents and their caregivers to return to complete the HPV vaccine series.

### Attitudes, beliefs, and experiences with HPV Vaccination Delivery

**Fig. 2:**
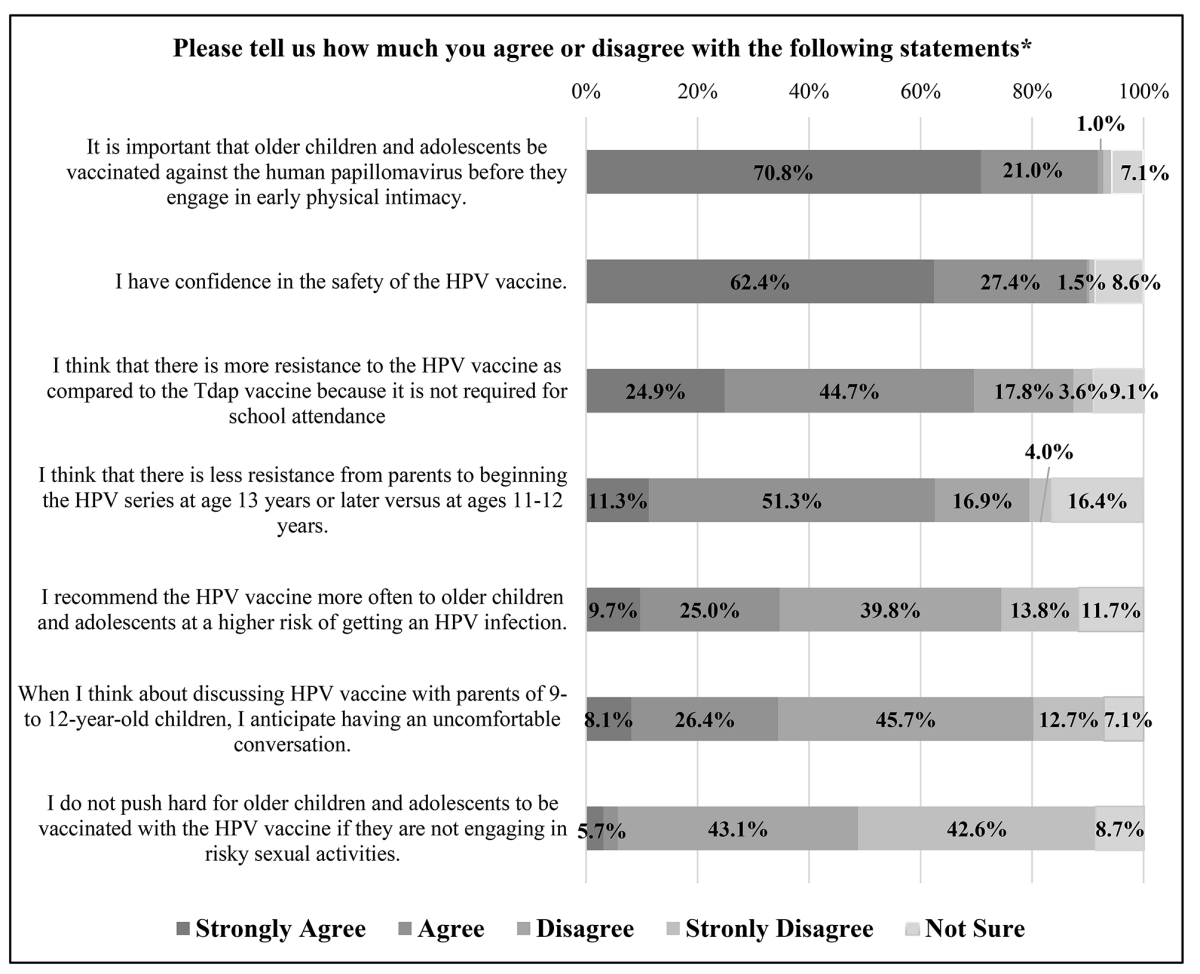
Nurses’ attitudes, beliefs, and experiences regarding human papillomavirus (HPV) vaccination for older children and adolescents. *percentages may not add to exact 100 due to rounding

About 91.8% (n = 179) of nurses agreed or strongly agreed that it was important that older children and adolescents be vaccinated against HPV before they engage in early physical intimacy, and a similar percentage (n = 177, 89.8%) expressed confidence in the safety of the HPV vaccine. However, about 34.5% (n = 68) of respondents reported anticipating an uncomfortable conversation while discussing the HPV vaccine with parents of 9 to 12-year-old children. Over two-thirds of respondents (n = 137, 69.6%) reported facing more resistance to the HPV vaccination as compared to the tetanus-diphtheria-acellular pertussis (Tdap) vaccine since Tdap vaccination is required by Montana state law for school attendance [13]. About 62.6% (n = 122) of nurses reported that parents prefer to initiate the HPV vaccine series for their children at 13 years or older versus at younger ages. Approximately one-third of nurses (n = 68, 34.7%) reported recommending the HPV vaccine more often to age-eligible adolescents at a higher risk of getting an HPV infection. (Fig. 2).

*Perceived barriers to HPV vaccine delivery*: More than two-thirds of nurses reported that they perceived the following as a major barrier or somewhat of a barrier to recommending and administering the HPV vaccine: parents not thinking that the vaccine is necessary for their sons (n = 146, 74.5%), misinformation that parents receive from the internet or social media (n = 139,71.6%), parental concerns about the safety of the HPV vaccine (n = 132, 67.7%), and irregular well-child visits (n = 130, 66.7%). Over half of respondents felt that the amount of time it takes to discuss HPV vaccination with parents or adolescents (n = 100, 51.3%) or the financial cost to get the HPV vaccine were not at all barriers to recommending or administering the HPV vaccine (n = 93, 47.5%). Through open-ended text box responses, nurses (n = 32,14.1%) reported additional barriers to the HPV vaccination, with the most frequently reported barriers being patient concerns about injection site pain, effects of the COVID-19 pandemic on regular well-child visits, and parental consent to receive the HPV vaccine. Respondents reported that parents of younger children (11-12-year-olds) were less aware that HPV vaccination is recommended for their child as compared to parents of older children (15-17-year-olds).

**Fig. 3:**
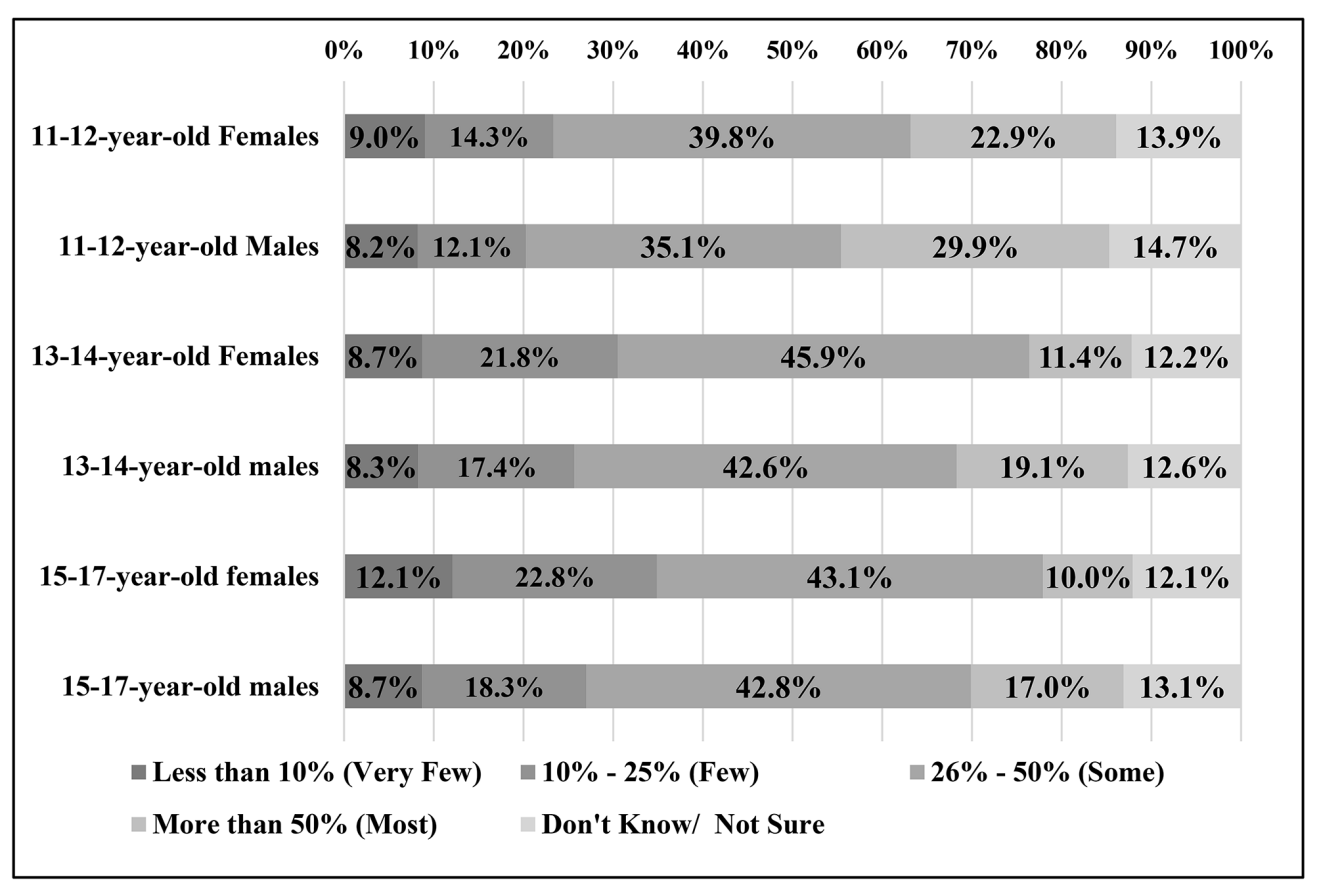
Nurses’ report of the estimated percentage of parents who defer HPV vaccination, by age group and sex of adolescent

A higher proportion of respondents reported over half of parental refusal or deferral of the HPV vaccine among younger age groups (11-12-year-olds) compared to older children (15-17-year-olds) regardless of the adolescent’s gender. (Fig. 3).

**Fig. 4:**
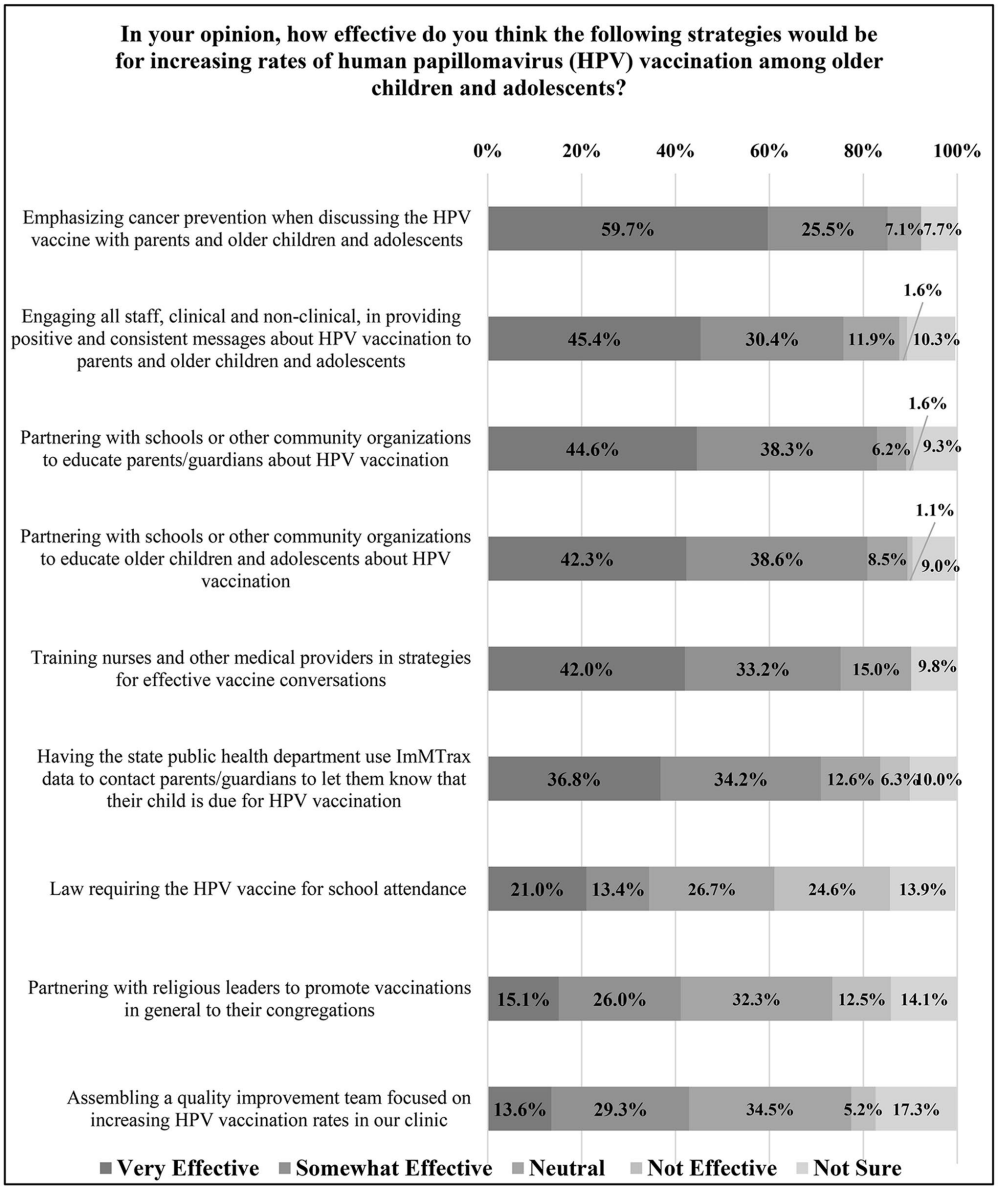
Nurses’ support of strategies to improve HPV vaccination rates

*Nurses’ support of strategies to improve HPV vaccination rates: Over three-fourths of nurses either strongly agreed or agreed that* emphasizing cancer prevention when discussing the HPV vaccine with parents and adolescents (n = 167,85.5%), partnering with a school or other community organizations in educating adolescents and parents about HPV vaccination (n = 160,82.9%), engaging all staff, clinical and non-clinical, in providing positive and consistent messages about HPV vaccination (n = 148,75.8%), training nurses and medical providers in strategies for effective vaccine conversations (n = 145,75.2%) were either very effective or somewhat effective strategies for increasing community HPV vaccination rates. Implementing a state law requiring the HPV vaccine for school attendance was least supported by the respondents (n = 68,34.4%) as a strategy to increase vaccine uptake. (Fig. 4) In open-ended responses, approximately 8.8% (n = 20) nurses provided additional ideas regarding initiatives to increase HPV vaccination including school-based vaccination clinics, incorporating immunizations within sports physicals, and providing education about the HPV vaccination through T.V. commercials or mailers.

## Discussion

Despite substantial evidence on the safety and efficacy of HPV vaccination in preventing oropharyngeal, anogenital, and other HPV-caused cancers, vaccine uptake is lower than established public health goals in Montana, particularly among rural adolescents. Prior research has highlighted the influential role that primary care providers, typically pediatricians and family medicine practitioners, play in promoting HPV vaccination [31, 32]. However, there has been less work focused on the important role of nurses in adolescent immunization services delivery efforts. Our study attempted to fill this significant gap in research related to nurses’ perceptions, experiences, and practices regarding HPV vaccination among older children and adolescents. Results from this study indicate that HPV vaccination was widely supported by Montana nurses, who also expressed confidence in the safety of the vaccine. Nurse respondents identified various barriers to HPV vaccine uptake in their communities, including parents’ lack of knowledge regarding which vaccines are recommended for adolescents, misinformation from social media, and specific parental concerns about the HPV vaccine. Importantly, nurses offered their input on strategies to increase vaccination uptake. Overall, the results from this survey point to several avenues for effectively engaging with nurses and medical assistants in HPV vaccination promotion efforts.

A provider’s strong recommendation is one of the most influential factors in parental decisions to get their children vaccinated against HPV [33]. However, about one-third of the survey respondents reported anticipating an uncomfortable conversation while discussing the HPV vaccine with adolescents or their parents. In our study findings, nurses indicated receptiveness toward training in effective vaccine conversations. Nationally, multiple studies on providers’ vaccine communication styles are linked with increased child and adolescent vaccine uptake, with a presumptive approach (i.e., assuming the parent intends to vaccinate) being more effective than a participatory style (i.e., asking if the parent would like to consider vaccination) [34–37]. There is also increasing attention toward motivational interviewing (MI) strategies that can be used to counsel vaccine-hesitant parents and address parents’ specific vaccine concerns. Motivational interviewing uses a collaborative conversation style to propel positive health behavior change and strengthen an individual’s motivation to change [38, 39]. The four motivational interviewing elements of open-ended questions, affirmations, reflection, and summary are built on the core principles of nursing practice which are connecting with the patient, evoking trust, and empathic listening [39]. A Swedish study focused on evaluating the proficiency of nurses in conducting motivational interviews with their patients indicated a need for nurses to receive additional training, feedback, and supervision in clinical practice with motivational interviewing techniques to achieve proficiency [40]. Empowering nurses to deliver strong vaccine recommendations and use MI techniques with vaccine-hesitant parents and caregivers may enable them to play a stronger role in increasing vaccination confidence and HPV vaccination uptake in their communities.

Reminder/recall systems are evidence-based practices that have been shown to increase vaccination rates among children, adolescents, and adults [28, 29]. The Task Force on Community Preventive Services recommends clinics perform some form of reminder-recall for their patients to improve vaccination rates [28]. In a narrative review about the contribution of reminder-recall systems to vaccine delivery efforts, Kempe et al. reported a 29% increase in adolescent vaccination rates at facilities that utilize reminder/recall processes [29]. In our study, nurses reported inconsistent use of reminder-recall systems for prompting parents to bring their children in when vaccines are due or past due. Previous studies analyzing providers’ perspectives have cited limited staff time, competing demands, insufficient technology, and increased costs as barriers to the successful implementation and sustenance of R/R systems [41, 42]. Providers in Montana, in which 90% of the counties are designated as health professional shortage areas [43], face similar challenges in their primary care practices which limit their ability to implement R/R processes. Innovative approaches such as centralized R/R systems may address the feasibility challenges of practice-based R/R in rural and medically underserved areas. Centralized R/R is conducted centrally either through healthcare systems or public health departments using an Immunization Information System [29]. Although large-scale implementation research studies evaluating the effects of centralized R/R systems on HPV vaccination rates only showed modest increases, these improvements could have a significant impact on reducing HPV-associated infections on a larger population level in the long run [44, 45]. Centralized R/R may be instrumental in reducing the burden on nursing professionals and awarding them more time to engage with parents and patients at their clinics.

Finally, the results from our survey indicate a need to increase community awareness about all four vaccines that are recommended for adolescents (Tdap, meningococcal, HPV, and influenza). Approximately 60.0% and 67.7% of survey respondents estimated that fewer than half of parents of 11-12-year-old females and males respectively were aware that the HPV vaccine was recommended for their children. Additionally, nurses also reported that parents’ refusal or deferral of the HPV vaccine was more common with 11-12-year-old children than with children ages 13 or older. Early marketing campaigns for the HPV vaccine were geared towards the prevention of sexually transmitted infections which caused discomfort among parents and providers while discussing the vaccine. Even though there has been a push to emphasize cancer prevention instead, safety concerns from parents and the public are still prevalent, owing to which parents have higher informational needs regarding the HPV vaccine relative to other vaccines [17]. Parents’ need for enhanced information and discussion may be a barrier to 11- or 12-year-old children getting the vaccine as recommended if this is the age when a trusted health professional first brings up the vaccine. Therefore, to increase on-time HPV vaccine uptake at ages 11–12 years, there is a need for widespread education of parents about recommended adolescent vaccines at earlier ages. National organizations like the American Cancer Society and the American Academy of Pediatrics have updated their recommendations to encourage providers to initiate the HPV vaccine series as early as 9 years of age [46, 47]. Starting the HPV vaccine series earlier at 9 years will likely lead to greater parental engagement and higher on-time vaccination rates. Providers will have the advantage of emphasizing cancer prevention while promoting the vaccine to their patients or parents and an opportunity to complete the series before the adolescent is due to receive school-entry required or other age-appropriate vaccines [46]. In this survey, many nurses reported being in favor of partnerships with schools or other community organizations to educate families about the HPV vaccine. Building community collaborations to leverage the reach of opinion leaders and social media in spreading positive messages about the HPV vaccination can help increase vaccine uptake [48, 49].

Our study findings align with findings from a qualitative study of healthcare personnel in rural Kentucky in suggesting that nursing professionals have a prominent role in assisting parents as they navigate health-related decisions for their children [50]. Future studies are needed to test these interventions among nurses to develop evidence-based communication strategies for nurses to effectively counsel and encourage parents and adolescents to receive the HPV vaccine. Nurses play a pivotal role in the ongoing efforts to increase awareness about the importance, safety, and effectiveness of the HPV vaccine [3]. As healthcare professionals who are readily accessible to families and adolescents, nurses are well-positioned to positively influence health behaviors and bring change to their communities [22].

Our study had some limitations. Firstly, our study was designed with descriptive objectives. Our aim was to provide a snapshot of nurses’ perceptions, experiences, and practices regarding human papillomavirus vaccination in a rural U.S. state. While we acknowledge that descriptive studies do carry a higher risk of selection and measurement biases, [51] we are confident that our study findings will have important public health implications in terms of improving nurses’ engagement with community promotion of HPV vaccination. We did not administer the survey to a fixed number of nurses and medical assistants. So, we were not able to compare the characteristics of respondents and non-respondents or produce a response rate. However, the metropolitan statistical area status-related distribution of 63.0% of rural nurses and 20.7% of urban nurses is representative of the geographic distribution of nursing professionals in Montana. Additionally, statistical power to compare responses across different professional groups was limited due to smaller sample sizes. Since this was a self-administered survey, the responses could be subjected to social desirability and recall bias. However, the anonymous nature of our survey may have reduced that likelihood. Finally, because our survey population consisted of nurses from Montana, the generalizability of the findings to nurses in other regions may be limited. However, given the urgent need to address persistently low HPV vaccination rates in rural areas of the U.S., this study of nurses and medical assistants in a predominately rural state adds to the limited previous research on engaging healthcare personnel in HPV vaccination promotion efforts in the rural U.S.

## Conclusion

In large, principally rural states like Montana, nurses and medical assistants play a key role in adolescent immunization delivery and often serve as the sole immunization providers in medically underserved areas. Because of the need to increase HPV vaccination rates to prevent HPV-caused cancers; the importance of providers’ vaccine recommendations; utilizing all clinic visits as opportunities to vaccinate; and understanding healthcare personnel’s knowledge, attitudes, and professional practices regarding the HPV vaccine are crucial for developing effective interventions focused on improving the consistency and strength of vaccine recommendations. Future studies should explore designing and employing novel approaches to tap into the potential of the existing workforce who are endowed with the required skills and harness their expertise in HPV vaccine promotion.

## Electronic supplementary material

Below is the link to the electronic supplementary material.

Supplementary Material 1

## Data Availability

The datasets generated and/or analyzed during the current study are not publicly available to protect the confidentiality of participants’ responses but are available from the corresponding author upon reasonable request.

## References

[1] Centers for Disease Control and Prevention. (2021, July 23). HPV infection. Centers for Disease Control and Prevention. Retrieved from https://www.cdc.gov/hpv/parents/about-hpv.html.

[2] Centers for Disease Control and Prevention. (2022, March 9). HPV vaccine-preventable diseases surveillance manual. Centers for Disease Control and Prevention. Retrieved from https://www.cdc.gov/vaccines/pubs/surv-manual/chpt05-hpv.html.

[3] Sherry JS, Collins SK, McKinnies RC, Fleege A, Walter ML (2018). Human papillomavirus and the nurse’s role in education and prevention. Health Care Manag.

[4] Meites E, Szilagyi PG, Chesson HW, Unger ER, Romero JR, Markowitz LE. Human papillomavirus vaccination for adults: updated recommendations of the advisory committee on immunization practices. Wiley; 2019. 10.15585/mmwr.mm6832a3 PMC6818701 31415491

[5] Williams CL, Walker TY, Elam-Evans LD (2020). Factors associated with not receiving HPV vaccine among adolescents by metropolitan statistical area status, united states, national immunization survey-teen, 2016–2017. Hum Vaccines Immunotherapeutics.

[6] Shipman SA, Lan J, Chang C, Goodman DC (2011). Geographic maldistribution of primary care for children. Pediatr (Evanston).

[7] Coombs NC, Campbell DG, Caringi J (2022). A qualitative study of rural healthcare providers’ views of social, cultural, and programmatic barriers to healthcare access. BMC Health Serv Res.

[8] Peterson CE, Silva A, Holt HK, Balanean A, Goben AH, Dykens JA (2020). Barriers and facilitators to HPV vaccine uptake among US rural populations: a scoping review. Cancer Causes Control.

[9] Daley MF, Crane LA, Markowitz LE (2010). Human papillomavirus vaccination practices: a survey of US physicians 18 months after licensure. Pediatrics.

[10] Allison MA, Hurley LP, Markowitz L (2016). Primary care physicians’ perspectives about the HPV vaccine. Pediatrics.

[11] Kempe A, O’Leary ST, Markowitz LE (2019). HPV vaccine delivery practices by primary care physicians. Pediatrics.

[12] Swiecki-Sikora AL, Henry KA, Kepka D. HPV vaccination coverage among US teens across the rural‐urban continuum. Wiley; 2019. 10.1111/jrh.12353 PMC6669111 30703854

[13] Zahnd WE, Rodriguez C, Jenkins WD (2019). Rural-urban differences in human papillomavirus‐associated cancer trends and rates. J Rural Health.

[14] Boyce TG, Christianson B, Hanson KE et al. Factors associated with human papillomavirus and meningococcal vaccination among adolescents living in rural and urban areas. 2022;11:100180. 10.1016/j.jvacx.2022.100180 PMC9218554 35755142

[15] U.S. Department of Health and Human Services. Increase the proportion of adolescents who get recommended doses of the HPV vaccine – IID-08. Office of Disease Prevention and Health Promotion. (n.d.). https://health.gov/healthypeople/objectives-and-data/browse-objectives/vaccination/increase-proportion-adolescents-who-get-recommended-doses-hpv-vaccine-iid-08.

[16] TeenVaxView. (2021, May 14). Centers for Disease Control and Prevention. Centers for Disease Control and Prevention. Retrieved December 11, 2022, from https://www.cdc.gov/vaccines/imz-managers/coverage/teenvaxview/data-reports/index.html.

[17] Newcomer SR, Caringi J, Jones B, Coyle E, Schehl T, Daley MF. (2020). A mixed-methods analysis of barriers to and facilitators of human papillomavirus vaccination among adolescents in Montana. Public Health Reports (1974), 135(6), 842–850. 10.1177/0033354920954512 PMC7649981 32972304

[18] Gilkey MB, McRee A (2016). Provider communication about HPV vaccination: a systematic review. Hum Vaccines Immunotherapeutics.

[19] Francis JKR, Rodriguez SA, Dorsey O (2021). Provider perspectives on communication and dismissal policies with HPV vaccine hesitant parents. Prev Med Rep.

[20] Bianco A, Mascaro V, Zucco R, Pavia M (2019). Parent perspectives on childhood vaccination: how to deal with vaccine hesitancy and refusal?. Vaccine.

[21] Deem MJ, Kronk RA, Staggs VS, Lucas D (2020). Nurses’ perspectives on the dismissal of vaccine-refusing families from pediatric and family care practices. Am J Health Promotion.

[22] Rosen BL, Ashwood D, Richardson GB (2016). School nurses’ professional practice in the HPV vaccine decision-making process. J School Nurs.

[23] McRee A, Gilkey MB, Dempsey AF (2014). HPV vaccine hesitancy: findings from a statewide survey of health care providers. J Pediatr Health Care.

[24] Roland KB, Benard VB, Greek A, Hawkins NA, Saraiya M (2014). Primary care providers human papillomavirus vaccine recommendations for the medically underserved: a pilot study in U.S. federally qualified health centers. Vaccine.

[25] Zimmerman RK, Nowalk MP, Mieczkowski TA, Mainzer HM, Jewell IK, Raymund M. (2001). The vaccines for children program Elsevier BV. 10.1016/s0749-3797(01)00359-2 11701292

[26] CDC (2014). VFC program continues to protect millions of children from disease. Infect Dis Child.

[27] Harris PA, Taylor R, Thielke R (2009). Research electronic data capture (REDCap) – a metadata-driven methodology and workflow process for providing translational research informatics support. J Biomed Inform.

[28] Jacobson Vann JC, Jacobson RM, Coyne-Beasley T (2018). Patient reminder and recall interventions to improve immunization rates. Cochrane Database Syst Rev.

[29] Kempe A, Stockwell MS, Szilagyi P (2021). The contribution of reminder-recall to vaccine delivery efforts: a narrative review. Acad Pediatr.

[30] Ryan K, Gannon-Slater N, Culbertson MJ (2012). Improving survey methods with cognitive interviews in small- and medium-scale evaluations. Am J Evaluation.

[31] Dorell C, Yankey D, Kennedy A, Stokley S (2013). Factors that influence parental vaccination decisions for adolescents, 13 to 17 years old: National Immunization Survey–Teen, 2010. Clin Pediatr.

[32] Kester LM, Zimet GD, Fortenberry JD, Kahn JA, Shew ML (2013). A national study of HPV vaccination of adolescent girls: rates, predictors, and reasons for non-vaccination. Maternal Child Health J.

[33] Oh NL, Biddell CB, Rhodes BE, Brewer NT. Provider communication and HPV vaccine uptake: a meta-analysis and systematic review. 2021;148:106554. 10.1016/j.ypmed.2021.106554 33857561

[34] Malo TL, Ali KN, Sutton SK, Perkins RB, Giuliano AR, Vadaparampil ST. (2016). The content and context of physicians’ communication with males about human papillomavirus vaccination Informa UK Limited. 10.1080/21645515.2015.1132963 PMC4964649 26835599

[35] Brewer NT, Hall ME, Malo TL, Gilkey MB, Quinn B, Lathren C. Announcements versus conversations to improve HPV vaccination coverage: a. randomized trial American Academy of Pediatrics (AAP); 1764. 10.1542/peds.2016-1764 PMC5192091 27940512

[36] Limaye RJ, Opel DJ, Dempsey A (2021). Communicating with vaccine-hesitant parents: a narrative review. Acad Pediatr.

[37] Reno JE, Thomas J, Pyrzanowski J (2019). Examining strategies for improving healthcare providers’ communication about adolescent HPV vaccination: evaluation of secondary outcomes in a randomized controlled trial. Hum Vaccines Immunotherapeutics.

[38] Koh-Knox CP (2009). Motivational interviewing in Health Care: helping patients change Behavior. Am J Pharm Educ.

[39] Breckenridge LA, Burns D, Nye C (2022). The use of motivational interviewing to overcome COVID-19 vaccine hesitancy in primary care settings. Public Health Nursing (Boston Massachusetts).

[40] Östlund A, Kristofferzon M, Häggström E, Wadensten B. Primary care nurses’ performance in motivational interviewing: a quantitative descriptive study. Springer Science and Business Media LLC; 2015. 10.1186/s12875-015-0304-z PMC4513379 26205692

[41] Saville AW, Albright K, Nowels C (2011). Getting under the hood: exploring issues that affect provider-based recall using an immunization information system. Acad Pediatr.

[42] Tierney CD, Yusuf H, McMahon SR (2003). Adoption of reminder and recall messages for immunizations by Pediatricians and Public Health Clinics. Pediatrics.

[43] Health Resources & Services Administration. Health Professional Shortage Area Find. 2021. Retrieved from https://data.hrsa.gov/tools/shortage-area/hpsa-find.

[44] Coley S, Hoefer D, Rausch-Phung E (2018). A population-based reminder intervention to improve human papillomavirus vaccination rates among adolescents at routine vaccination age. Vaccine.

[45] Gurfinkel D, Kempe A, Albertin C (2021). Centralized reminder/recall for human papillomavirus vaccination: findings from two States—A randomized clinical trial. J Adolesc Health.

[46] O’Leary ST, Nyquist AC. Why AAP recommends initiating HPV vaccination as early as age 9. Accessed July 18, 2022. American Academy of Pediatrics publications. 10.1080/21645515.2022.2146434 PMC9746363 36404635

[47] Saslow D, Andrews KS, Manassaram-Baptiste D, Smith RA, Fontham ETH (2020). Human papillomavirus vaccination 2020 guideline update: american cancer society guideline adaptation. Cancer J Clin.

[48] Vanderpool RC, Stradtman LR, Brandt HM (2019). Policy opportunities to increase HPV vaccination in rural communities. Hum Vaccines Immunotherapeutics.

[49] Bianco A, Della Polla G, Angelillo S, Pelullo CP, Licata F, Angelillo IF (2022). Parental COVID-19 vaccine hesitancy: a cross-sectional survey in Italy. Expert Rev Vaccines.

[50] Head KJ, Vanderpool RC, Mills LA (2013). Health care providers’ perspectives on low HPV vaccine uptake and adherence in Appalachian Kentucky. Public Health Nursing (Boston Mass).

[51] Aggarwal R, Ranganathan P (2019). Study designs: part 2. Descriptive Stud.

