# Supplementary material for: Nurses’ perceptions, experiences, and practices regarding human papillomavirus vaccination: results from a cross-sectional survey in Montana
Source: BMC Nurs. 2023 Jun 19;22:211. doi: 10.1186/s12912-023-01379-6 (PMC10278302; doi:10.1186/s12912-023-01379-6)
Supplement: Supplementary file 1 — Supplementary Material 1 [file 12912_2023_1379_MOESM1_ESM.pdf]

Please complete the survey below.

Thank you!

---

UNIVERSITY OF MONTANA
CENTER FOR POPULATION HEALTH RESEARCH

MONTANA DPHHS
Healthy People. Healthy Communities.
Department of Public Health & Human Services

---

The purpose of this survey is to learn about Montana nurses' and medical assistants' experiences with and perceptions of providing vaccines to older children and adolescents ages 9 to 17 years old. The survey is being conducted through a collaboration between the University of Montana Center for Population Health Research and the Montana Department of Public Health and Human Services, with funding from the Centers for Disease Control and Prevention. Your input will help inform public health initiatives and programs.

Your responses are important and will be kept confidential. There is minimal risk of a breach of confidentiality with your survey participation. We will not ask you to provide any identifiable information, and we will take multiple steps to protect the confidentiality of your survey responses, including securely storing survey data. In addition, we will only report survey findings in aggregate. While participating in this survey will provide no direct benefit to you, it will help in identifying areas for improving immunization services for adolescents in Montana.

At the end of the survey, you will be directed to a separate link where you can provide your name and email address to be entered into a drawing. We will randomly select four survey participants to receive a $30 Amazon gift card.

The survey takes approximately 15 minutes to complete. You can skip any questions that you prefer not to answer.

If you have any questions regarding this survey, you may contact Dr. Sophia Newcomer from the University of Montana's Center for Population Health Research at sophia.newcomer@umontana.edu or 406-243-4745.

---

| | |
|---|---|
| By clicking here, you consent to your voluntary participation in the survey. | ◯ Yes, I want to participate in this study<br>◯ No, I do not want to participate in this study |
| Do you currently work as a nurse or a medical assistant in the state of Montana? | ◯ Yes<br>◯ No |

| | |
|---|---|
| You indicated that you do not work as a nurse or a medical assistant in the state of Montana. Therefore, you are not eligible to complete this survey. We thank you for your interest.<br><br>Please select Yes to be re-directed to the end of the survey.<br><br>If this was an error and you want to return back to the survey, please select the Previous Page button below.<br><br>Have a nice day! | ◯ Yes |
| Please tell us about your involvement in immunization services for older children and adolescents.<br><br>Select all that apply. | ☐ I administer vaccines to older children and adolescents.<br>☐ I recommend vaccines to parents/guardians or patients.<br>☐ I answer parent's or patient's questions about vaccines.<br>☐ I schedule visits for immunizations.<br>☐ I am involved with ordering vaccines and managing vaccine inventory.<br>☐ I contact parents/guardians to let them know that their child is due or overdue for vaccines.<br>☐ I am involved with immunization services for older children and adolescents in other ways.<br>☐ I am not involved with immunization services for older children and adolescents. |
| Please tell us more about your involvement with immunization services for older children and adolescents. | __________________________________ |
| Survey 10% Complete | |
| You indicated that you are not involved with immunization services for older children or adolescents and therefore are not eligible to take the survey. We appreciate your interest.<br><br>Please select Yes to be redirected to the end of the survey.<br><br>If this was an error and you want to return back to the survey, please select the Previous Page button below.<br><br>Thank you! | ◯ Yes |

| | |
|---|---|
| What type of clinic setting do you work in? If you work across multiple clinics, for this survey, please focus on the clinic where you work most often.<br><br>Select all that apply. | ☐ Private practice<br>☐ Hospital-based clinic, including university clinic, or residency teaching practice<br>☐ Public health department-operated clinic<br>☐ Community health center<br>☐ Rural Health Clinic<br>☐ Migrant health center<br>☐ Indian Health Service (IHS)-operated center, Tribal health facility, or urban Indian health care facility<br>☐ Military health care facility (Army, Navy, Air Force, Marines, Coast Guard)<br>☐ WIC clinic<br>☐ School-based health center<br>☐ Other<br>☐ Not Sure |
| If you selected 'Private Practice' as your facility setting, please select the type of private practice. | ◯ Solo<br>◯ Group<br>◯ Health Maintenance Organization (HMO) |
| If you selected 'Other', please tell us the type of clinic you work for in the space provided. | __________________________________ |
| Please tell us about your clinic's medical specialty.<br><br>Select all that apply. | ☐ Pediatrics<br>☐ Family medicine<br>☐ Internal medicine<br>☐ Obstetrics and Gynecology<br>☐ Other |
| If you selected 'Other', then please specify your clinic's medical specialty in the space provided. | __________________________________ |
| What is your nursing or medical credential? | ◯ Licensed Practical Nurse (LPN)<br>◯ Registered Nurse (RN)<br>◯ Advanced Practice Registered Nurse (APRN)<br>◯ Medical Assistant<br>◯ Other |
| If you replied 'Other', please specify your nursing or medical credential in the space provided. | __________________________________ |
| You indicated that you are an Advanced Practice Registered Nurse (APRN). Please select your role. | ◯ Certified Nurse Practitioner<br>◯ Clinical Nurse Specialist<br>◯ Certified Registered Nurse Anesthetist<br>◯ Certified Nurse Midwife<br>◯ Other |
| If you replied 'Other', then please specify your role as an Advanced Practice Registered Nurse in the space provided. | __________________________________ |
| Do you currently work in direct patient care? | ◯ Yes<br>◯ No |

| | |
|---|---|
| On average, how many 9 to 17-year-old patients do you see as a nurse or a medical assistant in a typical week? | ◯ 0<br>◯ 1-5<br>◯ 6-10<br>◯ 11-20<br>◯ More than 20<br>◯ Not Sure |
| Does your facility participate in the Vaccines For Children (VFC) program? | ◯ Yes<br>◯ No<br>◯ Not Sure |
| Approximately what percentage of patients that you see are eligible for the Vaccines For Children (VFC) Program? | ◯ Less than 25%<br>◯ 25%-49%<br>◯ 50%-75%<br>◯ More than 75%<br>◯ Not Sure |
| Does your facility report vaccinations to ImMTrax? | ◯ Yes<br>◯ No<br>◯ Not sure |

Survey 20% Complete

SECTION 2: CLINIC VACCINATION PRACTICES

Next, we would like to ask about your clinic's routine practices for vaccinating older children and adolescents.

**Each of the following vaccines is approved for use in older children and adolescents ages 9-17 years.**

**In your clinic, which vaccines are routinely offered for the following age groups, if they are due?**

**Select all that apply**

| | Tetanus, diphtheria, and acellular pertussis (Tdap) vaccine | Influenza vaccine | Meningococcal vaccine | Human papillomavirus (HPV) vaccine |
|---|---|---|---|---|
| 9-10-year-old-patients | ☐ | ☐ | ☐ | ☐ |
| 11-12-year-old patients | ☐ | ☐ | ☐ | ☐ |
| 13-14-year-old patients | ☐ | ☐ | ☐ | ☐ |
| 15-17-year-old patients | ☐ | ☐ | ☐ | ☐ |

Survey 30% Complete

Does your clinic have a process to identify and contact parents/guardians of older children and adolescent patients who are due or overdue for immunizations?

- ◯ Yes
- ◯ No
- ◯ Not Sure

What is the process your clinic uses to identify and contact parents/guardians about immunizations that are due or past due?

Select all that apply.

- ☐ Reminder/recall list from ImMTrax
- ☐ An electronic report from the electronic medical record (EMR) system
- ☐ An electronic report from an administrative or billing system
- ☐ A paper-based system
- ☐ A tickler file, such as reminder cards that are tracked by nursing or administrative staff
- ☐ Reminder cards given to parents/guardians to remind them to return
- ☐ Other
- ☐ Not Sure

If you replied 'Other', then please specify the method used by your clinic to generate a list to contact parents/guardians about immunizations being due or past due in the space provided.

__________________________________________

How often are these tracking or reminder/recall lists generated?

- ◯ Weekly
- ◯ Monthly
- ◯ Our clinic generates these lists whenever there is staff capacity to do so.
- ◯ Other
- ◯ Not sure

If you replied 'Other', then please tell us how often these tracking or reminder/recall lists are generated in the space provided.

__________________________________

How does your clinic contact parents/guardians to let them know that vaccines are due or past due?

Select all that apply.

- ☐ Phone call
- ☐ Email
- ☐ Paper letter/Post card
- ☐ Text message
- ☐ Other
- ☐ Not sure

---

If you replied 'Other', then please tell us more about how your clinic contacts parents/guardians to let them know that vaccines are due or past due.

__________________________________

---

After the first HPV vaccine dose is administered, how do parents or patients in your clinic know to return for their next HPV vaccine dose?

Select all that apply.

- ☐ We tell them when the next dose is due and ask that they contact us to schedule a follow-up appointment.
- ☐ We schedule the follow-up visit before they leave the initial appointment.
- ☐ We contact them later to remind them to return for additional HPV doses.
- ☐ We use another method to remind them to return for additional HPV doses.
- ☐ We do not have a process for reminding parents or patients to return for additional HPV doses.

---

How does your clinic contact patients later on to return for their additional HPV vaccine doses?

- ☐ Phone Call
- ☐ Text Message
- ☐ E-mail
- ☐ Paper letter/ Post card

---

Please tell us the method your clinic uses to remind parents or patients to return for the additional HPV doses.

__________________________________

---

Survey 40% Complete

---

SECTION 3: NURSES' AND MEDICAL ASSISTANTS' EXPERIENCES & PERCEPTIONS

Now we want to ask about your experiences with older children and adolescent patients and their parents regarding their knowledge and acceptance of vaccines.

---

In Montana, 1 dose of tetanus-diphtheria-acellular pertussis (Tdap) vaccine is required prior to entering the 7th grade.

Based on your experiences, what proportion of parents are aware that vaccines other than Tdap are recommended to older children and adolescents?

- ○ Most (More than 75%) of the parents are aware that vaccines other than Tdap are recommended to older children and adolescents.
- ○ Some (About 50-75%) of the parents are aware that vaccines other than Tdap are recommended to older children and adolescents.
- ○ Few (About 25-49%) of the parents are aware that vaccines other than Tdap are recommended to older children and adolescents.
- ○ Very few (Less than 25%) of the parents are aware that vaccines other than Tdap are recommended to older children and adolescents.
- ○ Not sure

**In your experience, what percentage of parents/guardians are aware that the HPV vaccine is recommended in each of the following age groups?**

**One option can be chosen per row**

| | Less than 10% (Very Few) | 10% - 25% (Few) | 26% - 50% (Some) | More than 50% (Most) | Don't Know/Not Sure |
|---|---|---|---|---|---|
| 11-12-year-old females | ◯ | ◯ | ◯ | ◯ | ◯ |
| 11-12-year-old males | ◯ | ◯ | ◯ | ◯ | ◯ |
| 13-14-year-old females | ◯ | ◯ | ◯ | ◯ | ◯ |
| 13-14-year-old males | ◯ | ◯ | ◯ | ◯ | ◯ |
| 15-17-year-old females | ◯ | ◯ | ◯ | ◯ | ◯ |
| 15-17-year-old males | ◯ | ◯ | ◯ | ◯ | ◯ |

Survey 50% Complete

**In your experience, what percentage of parents and/or patients refuse or defer the HPV vaccine?**

**One option can be chosen per row**

| | Less than 10% (Very Few) | 10% - 25% (Few) | 26% - 50% (Some) | More than 50% (Most) | Don't Know/Not Sure |
|---|---|---|---|---|---|
| 11-12-year-old females | ◯ | ◯ | ◯ | ◯ | ◯ |
| 11-12-year-old males | ◯ | ◯ | ◯ | ◯ | ◯ |
| 13-14-year-old females | ◯ | ◯ | ◯ | ◯ | ◯ |
| 13-14-year-old males | ◯ | ◯ | ◯ | ◯ | ◯ |
| 15-17-year-old females | ◯ | ◯ | ◯ | ◯ | ◯ |
| 15-17-year-old males | ◯ | ◯ | ◯ | ◯ | ◯ |

Have you heard of the MT TeenVax Challenge?
◯ Yes
◯ No

Survey 60% Complete

**Please share your thoughts by answering the following questions about the MT TeenVax Challenge:**

| | Strongly Agree | Agree | Disagree | Strongly Disagree | Not Sure |
|---|---|---|---|---|---|
| I believe that the MT TeenVax Challenge has increased awareness about adolescent vaccinations among parents, older children, and adolescents in my clinic. | ○ | ○ | ○ | ○ | ○ |
| I believe that continuing the MT TeenVax Challenge will help boost adolescent immunization rates in my clinic. | ○ | ○ | ○ | ○ | ○ |
| I think that the MT TeenVax Challenge helps promote adolescent vaccines in rural or underserved areas of Montana. | ○ | ○ | ○ | ○ | ○ |

Survey 60% Complete

**Based on your experiences as a nurse or a medical assistant, are any of the following barriers to older children and adolescents 9-17 years of age receiving the human papillomavirus (HPV) vaccine?**

**One option can be chosen per row:**

| | A Major Barrier | Somewhat of a Barrier | A Minor Barrier | Not at All a Barrier | Don't Know/Not Sure |
|---|---|---|---|---|---|
| Misinformation parents receive from the Internet or social media regarding the HPV vaccine | ○ | ○ | ○ | ○ | ○ |
| Parental concerns about the safety of the HPV vaccine | ○ | ○ | ○ | ○ | ○ |
| Parents not thinking that the HPV vaccine is necessary for their sons | ○ | ○ | ○ | ○ | ○ |
| Parents not thinking that the HPV vaccine is necessary for their daughters | ○ | ○ | ○ | ○ | ○ |
| Parental concerns that HPV vaccination may encourage their child to have earlier sexual behavior | ○ | ○ | ○ | ○ | ○ |
| Parental concerns about giving too many vaccines in one visit | ○ | ○ | ○ | ○ | ○ |
| Lack of school requirement for the HPV vaccine | ○ | ○ | ○ | ○ | ○ |
| The amount of time it takes to talk about the HPV vaccine with parents and patients | ○ | ○ | ○ | ○ | ○ |
| Parent's moral opposition to the HPV vaccine | ○ | ○ | ○ | ○ | ○ |
| Medical providers, such as physicians, not recommending the HPV vaccine | ○ | ○ | ○ | ○ | ○ |
| Financial cost to get the HPV vaccine | ○ | ○ | ○ | ○ | ○ |
| Older children and adolescents not coming to the clinic for regular well-child visits | ○ | ○ | ○ | ○ | ○ |

In the space provided, please describe any other barriers you have seen to older children and adolescents 9-17 years of age receiving the HPV vaccine. ______________________________

Survey 70% Complete

SECTION 4: VACCINE ATTITUDES AND BELIEFS

Now, we would like to learn about your thoughts on vaccines for older children and adolescents.

**Please tell us how much you agree or disagree with the following statements. Please mark only one option per statement.**

| | Strongly Agree | Agree | Disagree | Strongly Disagree | Not Sure |
|---|---|---|---|---|---|
| It is important that older children and adolescents be vaccinated against the human papillomavirus before they engage in early physical intimacy. | ○ | ○ | ○ | ○ | ○ |
| When I think about discussing HPV vaccine with parents of 9- to 12-year-old children, I anticipate having an uncomfortable conversation. | ○ | ○ | ○ | ○ | ○ |
| I think that there is more resistance to the HPV vaccine as compared to the Tdap vaccine because it is not required for school attendance. | ○ | ○ | ○ | ○ | ○ |
| I think that there is less resistance from parents to beginning the HPV series at age 13 years or later versus at ages 11-12 years. | ○ | ○ | ○ | ○ | ○ |
| I recommend the HPV vaccine more often to older children and adolescents at a higher risk of getting an HPV infection. | ○ | ○ | ○ | ○ | ○ |
| I do not push hard for older children and adolescents to be vaccinated with the HPV vaccine if they are not engaging in risky sexual activities. | ○ | ○ | ○ | ○ | ○ |
| I have confidence in the safety of the HPV vaccine. | ○ | ○ | ○ | ○ | ○ |

Survey 80% Complete

**In your opinion, how effective do you think the following strategies would be for increasing rates of human papillomavirus (HPV) vaccination among older children and adolescents?**

| | Very Effective | Somewhat Effective | Neutral | Not Effective | Don't Know/Not Sure |
|---|---|---|---|---|---|
| Emphasizing cancer prevention when discussing the HPV vaccine with parents and older children and adolescents | ○ | ○ | ○ | ○ | ○ |
| Assembling a quality improvement team focused on increasing HPV vaccination rates in our clinic | ○ | ○ | ○ | ○ | ○ |
| Training nurses and other medical providers in strategies for effective vaccine conversations | ○ | ○ | ○ | ○ | ○ |
| Engaging all staff, clinical and non-clinical, in providing positive and consistent messages about HPV vaccination to parents and older children and adolescents | ○ | ○ | ○ | ○ | ○ |
| Law requiring the HPV vaccine for school attendance | ○ | ○ | ○ | ○ | ○ |

**Question Continued**

**In your opinion, how effective do you think the following strategies would be for increasing rates of human papillomavirus (HPV) vaccination among older children and adolescents?**

| | Very Effective | Somewhat effective | Neutral | Not Effective | Don't Know/Not Sure |
|---|---|---|---|---|---|
| Partnering with schools or other community organizations to educate parents/guardians about HPV vaccination | ○ | ○ | ○ | ○ | ○ |
| Partnering with schools or other community organizations to educate older children and adolescents about HPV vaccination | ○ | ○ | ○ | ○ | ○ |
| Partnering with religious leaders to promote vaccinations in general to their congregations | ○ | ○ | ○ | ○ | ○ |
| Having the state public health department use ImMTrax data to contact parents/guardians to let them know that their child is due for HPV vaccination | ○ | ○ | ○ | ○ | ○ |

Please list any other strategies that would be effective in increasing HPV vaccination rates among older children and adolescents in Montana. ______________________________

Survey 90% Complete

SECTION 5: FINAL QUESTIONS

Thank you for taking this survey. Your input is important and valuable. We just have a few more questions.

Which county or reservation do you work on?

◯ Beaverhead
◯ Big Horn
◯ Blackfeet Reservation
◯ Blaine
◯ Broadwater
◯ Carbon
◯ Carter
◯ Cascade
◯ Chouteau
◯ Crow Reservation
◯ Custer
◯ Daniels
◯ Dawson
◯ Deer Lodge
◯ Fallon
◯ Fergus
◯ Flathead
◯ Flathead Reservation
◯ Fort Belknap Reservation
◯ Fort Peck Reservation
◯ Gallatin
◯ Garfield
◯ Glacier
◯ Golden Valley
◯ Granite
◯ Hill
◯ Jefferson
◯ Judith Basin
◯ Lake
◯ Lewis and Clark
◯ Liberty
◯ Lincoln
◯ Madison
◯ McCone
◯ Meagher
◯ Mineral
◯ Missoula
◯ Musselshell
◯ Northern Cheyenne Reservation
◯ Park
◯ Petroleum
◯ Phillips
◯ Pondera
◯ Powder River
◯ Powell
◯ Prairie
◯ Ravalli
◯ Richland
◯ Rocky Boy's Reservation
◯ Roosevelt
◯ Rosebud
◯ Salish and Kootenai Reservation
◯ Sanders
◯ Sheridan
◯ Silver Bow
◯ Stillwater
◯ Sweet Grass
◯ Teton
◯ Toole
◯ Treasure
◯ Valley
◯ Wheatland
◯ Wibaux
◯ Yellowstone

| | |
|---|---|
| For how many years have you worked as a nurse or a medical assistant? | ◯ Less than 2 years<br>◯ 2-6 years<br>◯ 6-10 years<br>◯ 11-15 years<br>◯ 16-20 years<br>◯ More than 20 years<br>◯ Prefer not to answer |
| What is your sex? | ◯ Female<br>◯ Male<br>◯ Other<br>◯ Prefer not to answer |
| How old are you? | ◯ Less than 20 years<br>◯ 21- 30 years<br>◯ 31-40 years<br>◯ 41- 50 years<br>◯ 51- 60 years<br>◯ ≥61 years<br>◯ Prefer not to answer |
| Are you of Hispanic or Latino origin? | ◯ Yes<br>◯ No<br>◯ Prefer not to answer |
| How would you describe yourself? | ☐ American Indian or Alaska Native<br>☐ Asian<br>☐ Black or African American<br>☐ Native Hawaiian or Other Pacific Islander<br>☐ White<br>☐ Other<br>☐ Prefer not to answer |
| Thank you for participating in this survey. Please use this text box to share any other comments or suggestions regarding immunization practices and strategies for older children and adolescents. | ______________________________ |

This is the end of the survey. We thank you for your time and input. Please make sure to submit your responses using the submit button below before exiting the survey.
